# Computed Tomographic Image Analysis Based on FEM Performance Comparison of Segmentation on Knee Joint Reconstruction

**DOI:** 10.1155/2014/235858

**Published:** 2014-11-27

**Authors:** Seong-Wook Jang, Young-Jin Seo, Yon-Sik Yoo, Yoon Sang Kim

**Affiliations:** ^1^Department of Computer Science and Engineering, Korea University of Technology and Education, 330-708 Cheonan, Republic of Korea; ^2^Department of Orthopaedic Surgery, Hallym University, 446-907 Dongtan, Republic of Korea

## Abstract

The demand for an accurate and accessible image segmentation to generate 3D models from CT scan data has been increasing as such models are required in many areas of orthopedics. In this paper, to find the optimal image segmentation to create a 3D model of the knee CT data, we compared and validated segmentation algorithms based on both objective comparisons and finite element (FE) analysis. For comparison purposes, we used 1 model reconstructed in accordance with the instructions of a clinical professional and 3 models reconstructed using image processing algorithms (Sobel operator, Laplacian of Gaussian operator, and Canny edge detection). Comparison was performed by inspecting intermodel morphological deviations with the iterative closest point (ICP) algorithm, and FE analysis was performed to examine the effects of the segmentation algorithm on the results of the knee joint movement analysis.

## 1. Introduction

Recent developments and the greater availability of computer technology have enabled much actively conducted research combining digital imaging technologies such as CT and MRI with finite element (FE) analysis. This type of research has been increasing, particularly in the field of orthopedics, as the knee joint is relatively easy to model.

Also, a variety of studies have verified anatomical interpretations and performed sports rehabilitation simulations by examining stress distributions caused by movements of the knee.

Kim et al. [1] established a model exactly simulating the knee joint movement of a real patient by changing a bone model built from four different angles in a three-dimensional FE model and analyzed the results of stress distribution in the matching anterior cruciate ligament (ACL) graft. Yagi et al. [2], Hirokawa and Tsuruno [3], and Hame et al. [4] validated the single-bundle reconstruction technique using FE analysis and Li et al. [5] analyzed ACL movement (extension, rotation, and twist) induced by knee-flexion movement. Peña et al. [6] analyzed changes in ACL stress depending on pretension strength and knee angle.

Moreover, some studies have analyzed changes in ACL stress by focusing on the pretension of various steps and knee angles and then analyzing the stress distributions for ACL reconstruction by applying the same movements as that of the patients. Subsequently, they reconstructed knee bones from four angles by using a 3D FE model.

The model used in the research described above was a 3D bone model created from CT data and used for analysis under the assumption that ACL transplant grafts are cylindrical. To create a solid model of the body from CT imaging, an image processing method was used to segment a region of interest from a 2-dimensional (2D) image of the bone. The 3D solid model was constructed by interpolating the slices made by stacking separate regions based on the image processing methods.

Therefore, clinicians must accurately segment edges. For segmentation of an exact region, the help of skilled expert was required, and it became problematic that the accuracy differed according to the expert's proficiency. In order to solve this problem, a variety of image processing algorithms were applied.

The methods of applying a segmentation algorithm to a region of interest were investigated by applying the Sobel operator [7–9] and the Laplacian of Gaussian (LoG) operator [9–12] and by using Canny edge detection [13–15].

Antonelli et al. [7] applied combination of image processing techniques for the automated identification of the pulmonary volume. Magnusson et al. [8] investigated the shaded surface display of radiological 3D density volumes from the twelve different cases applied on both the ideal sphere and a real human skull. A framework for segmentation of cerebral vessel images was defined and validated in [9]. Ecabert et al. [10] introduced a 3D model-based approach for the segmentation of cardiac CT images including four chambers, myocardium, and great vessels. Conte et al. [11] proposed a new segmentation method for MRI images by integrating region growing with level set segmentation. Bomans et al. [12] showed that a combination of an edge detection operator and morphological filters for the refinement of the detected edges could detect surfaces of organs in MRI volumes reasonably well. Rathnayaka et al. [13] investigated the performance on both intensity thresholding and Canny edge detection methods used to segment image data for generating 3D models of long bones from CT scanning of cadaver sheep specimens. The MRI- and CT-based 3D models of the femora of cadaver ovine limbs were compared and validated against the reference models [14]. Punarselvam and Suresh [15] improved Canny edge detection and used preprocessing of boundary detection of the CT scan Spine disc image.

While theoretical comparative image processing algorithm research has been conducted, there has not been any research to date to determine the optimal algorithm to create a 3D model from human CT images.

This study aims to find the most suitable segmentation algorithm to reconstruct a 3D model from the knee (joint) CT images. To achieve this goal, we compare and validate segmentation algorithms based on both objective comparisons and FE analysis. Stress distributions are compared and analyzed for reconstructed 3D models by examining morphological errors and FE analysis using edge segmentation algorithms typically used in the field of image processing. First, we analyze the Sobel operator, LoG operator, and the Canny edge detection algorithm, which are commonly used to compare CT image segmentation performance. Additionally, we determine the reliability of each algorithm by comparing the model constructed using image processing algorithms to a model constructed manually by clinicians. Next, we compare the performance of each algorithm by using FE analysis of the segmented reconstructed ACL.

## 2. Materials and Methods

### 2.1. Segmentation Algorithms for Knee Model Reconstruction

FE modeling for ACL analysis was created based on the CT data of the experimenter. CT data was provided in Digital Imaging and Communications in Medicine (DICOM), a format typically used in medical imaging and showed the internal biometric information as a single slice 1 to 2 mm in thickness. To reconstruct a 3D knee model, extracting the region from the acquired CT data is necessary, and this process is called segmentation. Stacked contours of segmented regions are used to create a 3D surface model through interpolation. This paper uses the Sobel operator, Laplacian of Gaussian operator, and Canny edge detection algorithms which are widely used in the medical image processing [16, 17].

#### 2.1.1. Manual Edge Segmentation by an Expert (Method 1)

The simplest segmentation method is for clinicians to mark the region themselves. Knee joint CT data scanned in 2 mm intervals can result in more than 200 total slices (100 slices each of the femur and tibia), and the segmented area should be delineated manually to reconstruct a 3D knee model. This method depends on the knowledge, proficiency, and condition of the person who is delineating the region and has the disadvantage that the accuracy of the created model depends on individual proficiency.

#### 2.1.2. Sobel Operator Algorithm (Method 2)

The Sobel operator used for boundary detection is very sensitive for the boundary towards diagonal direction. The Sobel operator determines the magnitude of the gradient using *G*_*x*_, horizontal gradient component, and *G*_*y*_, vertical gradient component, as shown in

(1) $$\begin{array}{c} G=\sqrt{G_{x}^{2}+G_{y}^{2}}, \end{array}$$

where

(2) Gx=−1−2−1000121∗A,Gy=−101−202−101∗A

(*A* is original image and *G*_*x*_ and *G*_*y*_ are horizontal and vertical orientation kernels of gradient).

It is difficult to detect a detailed contour as the size of the kernel gets larger, whereas it becomes sensitive to noise as the one gets smaller.

This differential operator generally tends to be very sensitive to noise, resulting in malfunction by considering the noise pixel as an edge.

#### 2.1.3. Laplacian of Gaussian Operator Algorithm (Method 3)

The Laplacian of Gaussian (LoG) operator is an algorithm, upgraded from Laplacian operator, that detects blob structures surrounded with the area separated by the level of the nearby pixels. The LoG operator is the second derivative edge detector and is less sensitive to noise. It can be expressed as the sum of the second derivative functions towards the horizontal and vertical orientation of gradient. The LoG operator performs Gaussian smoothing before applying the Laplacian filter. The Laplacian operator obtains the edge component on the first derivate inflection point where the second derivative is a zero and is sensitive to the noise on the point where the second derivative is obtained. LoG operator improves the robustness for the noise by applying Gaussian operator, as shown in (3).

The filter of the LoG operator is determined by the value of Gaussian standard deviation (*σ*), which affects the smoothing of the image. Consider

(3) $$\begin{array}{c} \text{LoG}\left(x,y\right)=\frac{1}{\pi \sigma^{4}}\left[1-\frac{x^{2}+y^{2}}{2\sigma^{2}}\right]e^{-\left(x^{2}+y^{2}\right)/2\sigma^{2}}, \end{array}$$

where *σ* is Gaussian standard deviation.

#### 2.1.4. Canny Edge Detection Algorithm (Method 4)

The Canny edge detection algorithm can be used as an optimal edge detector based on a set of criteria, which include finding the most edges by minimizing the error rate, marking edges as closely as possible to the actual edges to maximize localization, and marking edges only once when a single edge exists for a minimal response. Also, the result detected by Canny edge detection algorithm should reduce the loss of edge component and the error between the detected edge and the real gradient on original image. According to Canny, the optimal filter that meets all 3 criteria above can be efficiently approximated using the first derivative of a Gaussian function by (4).

The Gaussian filter is determined by the value of standard deviation (*σ*) and affects the smoothing of the image. Once the size of the filter is determined, the image by the Gaussian smoothing can be obtained. Sensitivity to noise depends on the size of the filter; the sensitivity is decreased as the size of the filter is increased. Consider

(4) $$\begin{array}{c} G\left(x,y\right)=\frac{1}{2\pi \sigma^{2}}e^{-\left(x^{2}+y^{2}\right)/2\sigma^{2}}, \end{array}$$

where *σ* is Gaussian standard deviation.

(1) Calculate the average magnitude. Consider
  (5) $$\begin{array}{c} M\left(x,y\right)=\frac{1}{M}\overset{n}{\underset{1}{\sum }}\sqrt{{M_{x}\left(x,y\right)}^{2}+{M_{y}\left(x,y\right)}^{2}}, \end{array}$$
 where *M*_*x*_ and *M*_*y*_ are the average magnitudes of the horizontal and vertical gradient, respectively.
(2) Calculate the density of the edge length. The density of the edge length is calculated from
  (6) $$\begin{array}{c} L\left(x,y\right)=\frac{C\left(x,y\right)}{\max C\left(x,y\right)}, \end{array}$$
 where *C*(*x*, *y*) is the number of connected pixels for each pixel position.

**Figure 1:**
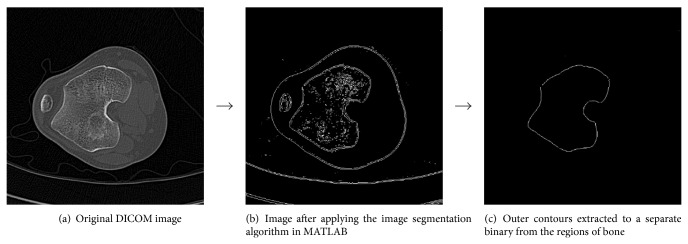
Segmentation procedure for the knee joint by the Sobel operator algorithm.

**Figure 2:**
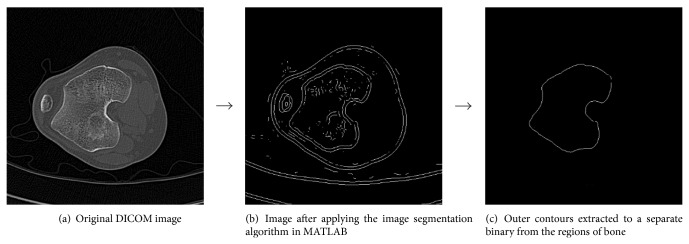
Segmentation procedure for the knee joint by the Laplacian of Gaussian operator algorithm (where input parameters *σ* = 4).

**Figure 3:**
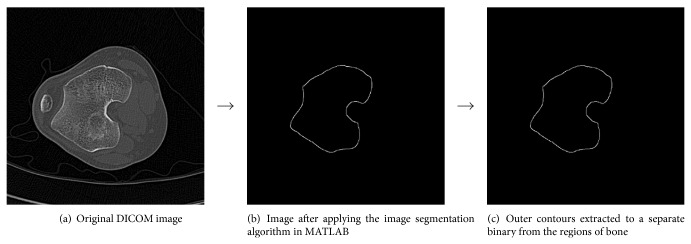
Segmentation procedure for the knee joint by the Canny edge detection algorithm (where input parameters *σ* = 2, low threshold = 0.10, and high threshold = 0. 90).

The segmentation process using the three algorithms as described above is shown in Figures 1, 2, and 3, respectively.

### 2.2. Experimental Methods

In vivo measurements were made in a 27-year-old man with no history of knee pathology or injury. The CT scan and data acquisition were performed on the right knee at the Hallym University Medical Center, Dongtan, Korea [18]. The subject's right knee was scanned in the lateral decubitus position with a high-resolution CT scanner [19] with 1 mm thick slices obtained at four different knee flexion angles (0°, 45°, 90°, and 135°) in motions ranging from 0° to 135°.

**Figure 4:**
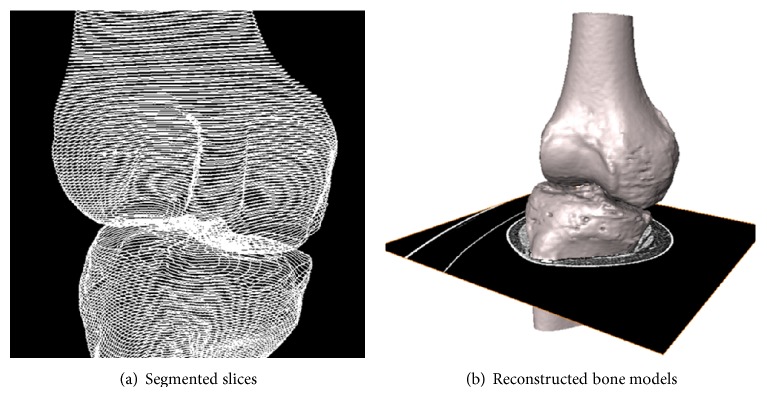
Knee model reconstruction using interpolation.

In this part, we compared each model shape by applying four types of algorithms to the 3D knee joint model created from CT image data and confirmed the effect of the segmentation algorithm result with FE analysis of ACL reconstruction surgery. We used three types of image processing algorithms (the Sobel operator, LoG operator, and Canny edge detector) for bone model segmentation. Additionally, we compared the models reconstructed using each algorithm to the model manually segmented by clinicians. The reconstructions performed using manual clinician segmentation, the Sobel operator, the LoG operator, and the Canny edge detection algorithms are described as Methods 1, 2, 3, and 4, respectively. Stacked contours (Figure 4(a)) from the applied segmentation algorithm were interpolated and used to construct a surface model (Figure 4(b)).

**Figure 5:**
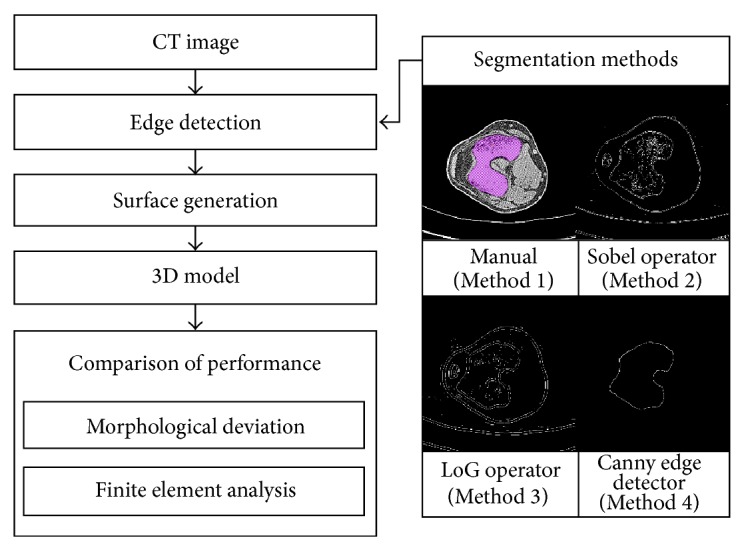
Experimental procedure.

Image processing algorithms were applied to CT images using MATLAB [20]. A knee model was constructed from the segmented regions by using Amira [21]. Rapidform [22] was used to construct the tunnel in the ACL graft model and to compare morphological deviations. Abaqus/Explicit code [23] was used to construct the FE model and for analysis.  Figure 5 shows the experimental procedure for this study.

#### 2.2.1. Comparison on Morphological Deviations of Reconstructed Models

The models reconstructed by each algorithm show different morphological errors according to the segmented area. Three-dimensional morphological deviations between the models are obtained and analyzed by comparing the distances between the mesh of each model.

Morphological deviations were obtained to match points between Method 1 and Methods 2, 3, and 4. 3D surface matching was performed using the ICP algorithm [24], which seeks to minimize the sum of the squared distances between all points on the source surface and their closest points on the target model surface.

#### 2.2.2. Comparison on Simulations of Reconstructed Models Using Finite Element Analysis

**Figure 6:**
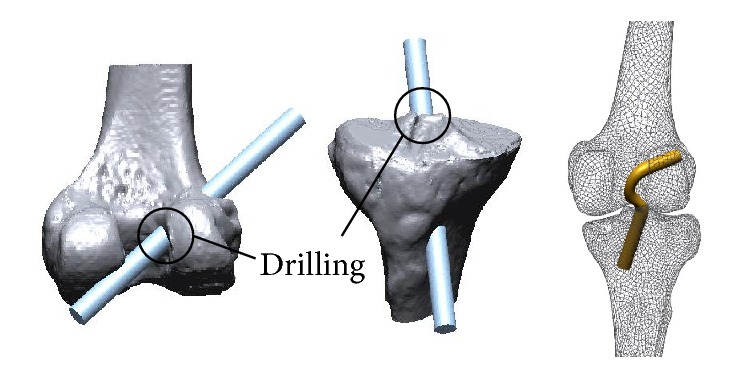
Construction of the tunnel and ACL graft.

In this section, we explain the process of generating a FE model, which was applied to the four segmentation algorithms to compare the effects of FE analysis. To generate a solid model with the same tunnel as is made in a real operation of the femur and tibia, 10 mm diameter tunnels were drilled in the center between the anteromedial (AM) bundle and the posterolateral (PL) bundle, as shown in Figure 6. The cylinder 9 mm in diameter at the center of the tunnel penetrating the solid model is the ACL bundle model. The Fillet function was applied to provide smooth contact between the ligament and bone.

**Figure 7:**
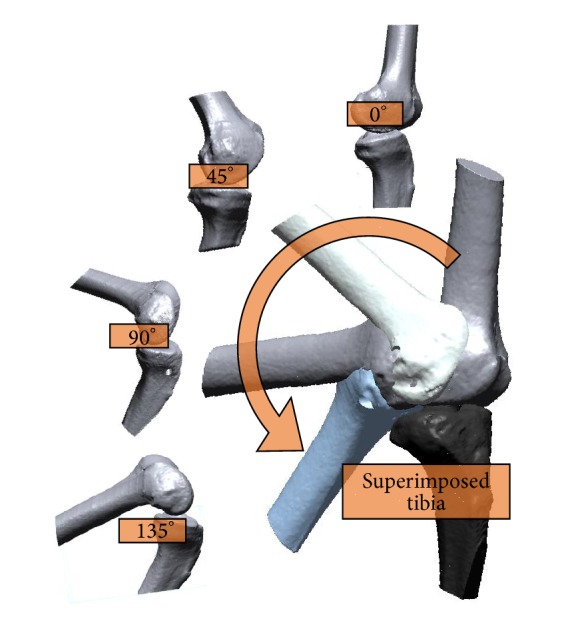
Determining the superimposed position of the tibia.

It is not easy to estimate the trajectories of knee joint movement because the human knee does not have a specific center of rotation. To overcome this difficulty, knee joint angle trajectories were mapped by imaging them in several positions while the experimental subject was executing knee movements. The simulations of the reconstructed models were then compared using the images obtained from four different angles (0°, 45°, 90°, and 135°) and the locations of the femur in each angle were superimposed based on the tibia. The superimposed femur was ensured by the Align function in Rapidform; the results are shown in Figure 7.

**Figure 8:**
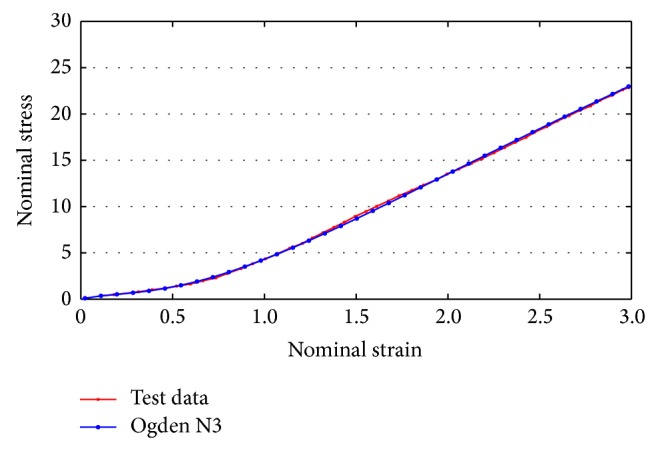
Curve fitting using a hyperelastic material model of ACL.

The FE model consists of a mesh, and the femur and tibia were assumed to be rigid bodies considering computational burden and analysis time. The ACL was assumed to be a hyperelastic material. A hyperelastic material property is generally used in modeling rubber with very large deformations. The material characteristics of the bundles of the reconstructed ACL are expressed as (9), characterized by a strain energy potential function such as in an Ogden model, and Figure 8 shows the stress-strain relationship and curve fitting using the hyperelastic material model [25].

From classical continuum mechanics, the right Cauchy-Green deformation tensor is defined as

(7) $$\begin{array}{c} C=F^{T}F, \end{array}$$

where *F* is the deformation gradient.

The Ogden model is a hyperelastic material model, which can describe the material behavior by means of the strain energy density function [26]:

(8) $$\begin{array}{c} W_{O}=\overset{N}{\underset{i=1}{\sum }}\frac{\mu_{i}}{\alpha_{i}}\left[\lambda_{1}^{\alpha_{i}}+\lambda_{2}^{\alpha_{i}}+\lambda_{3}^{\alpha_{i}}-3+\frac{1}{d_{i}}{\left(J-1\right)}^{2i}\right], \end{array}$$

where *N* is the model's order, *α*_*i*_ and *μ*_*i*_ are the parameters to be determined experimentally, and *d*_*i*_ is the volume change parameter. *λ*_*k*_ (*k* = 1,2, 3), also called principal stretches, refer to the eigenvalues of the deformation tensor *C*. *J* refers to the Jacobian of the deformation gradient.

Because the high water content of ACL is assumed to be incompressible, that is, the Jacobian of the deformation gradient, *J* = *λ*_1_ *λ*_2_ *λ*_3_ = 1. Equation (8) can then be simplified to (9) as below:

(9) $$\begin{array}{c} W_{O}=\overset{N}{\underset{i=1}{\sum }}\frac{\mu_{i}}{\alpha_{i}}\left[\lambda_{1}^{\alpha_{i}}+\lambda_{2}^{\alpha_{i}}+\lambda_{3}^{\alpha_{i}}-3\right]. \end{array}$$

Then we can have the principal stress *σ*_*k*_ = *λ*_*k*_(∂*W*_*O*_/∂*λ*_*k*_).

Mesh cannot be generated by only hexahedrons because bone is composed of curved surfaces. For this reason, the bones were generated as tetrahedrons, and the ACL model was generated as hexahedrons. The femur and tibia were modeled using a large number of linear tetrahedral elements (C3D4). The total number of nodes and elements for the femur was 16577 and 84018, respectively, and the total number of nodes and elements for the tibia was 16221 and 80274, respectively. The ACLs were modeled using a large number of linear hexahedral elements (C3D8R). The total number of nodes and elements for the ACL was 11832 and 10005, respectively. Boundary conditions, including load, contact, and tie, should be applied to perform the FE analysis of knee flexion. The loading condition was divided into two steps. In the first step, the translation and rotation of the tibia's 6-DOF (*x*-, *y*-, and *z*-axes and pitch, roll, and yaw) were fixed. In the second step, a sequential movement of femoral flexion was reproduced using femur models at different angles (0, 45, 90, and 135 degrees) based on the fixed tibia. The frictional coefficients of the penalty formula were set at 0.1 for the bone-ligament contacts [25]. The femur-ACL tie and the tibia-ACL tie were fixed at the same distance (10 mm). The AM and PL bundles were put in each tunnel under 40 N of tension and the grafts were fixed.

## 3. Results and Discussion

### 3.1. Morphological Comparisons on Models Generated from Segmentation Algorithms

**Figure 9:**
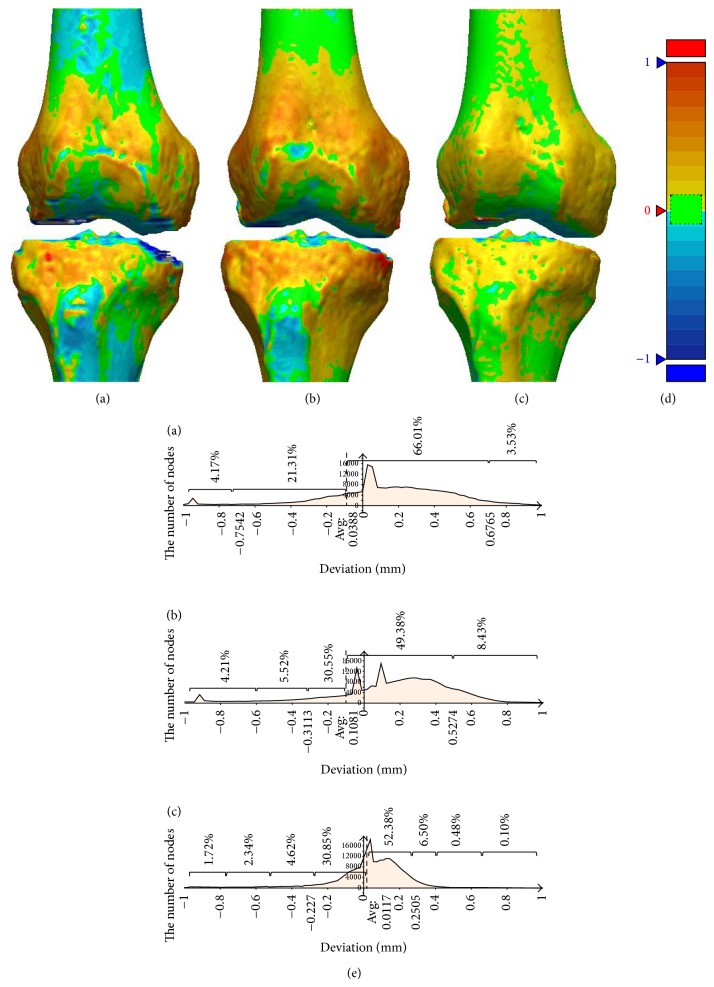
Morphological comparison on two bones. (a) Shape comparisons for Methods 1 and 2, (b) Methods 1 and 3, (c) Methods 1 and 4, (d) the spectrum of colour map, and (e) morphological deviations distribution for ((a), (b), and (c)). (Dimensions larger than those of Method 1 model are indicated by the color red; smaller dimensions are represented by blue. When the models are similar and the morphological deviation approaches 0 mm, they are described as green.)

Figure 9 shows the results of the morphological deviation comparison for each algorithm, based on reference model (Method 1). Figures 9(a), 9(b), and 9(c) show the distance deviations between the reference model (Method 1) reconstructed by a clinical expert and the models reconstructed by 3 algorithms (Methods 2, 3, and 4) by color map. The color map is described by the spectrum (−1 mm~1 mm) as shown in Figure 9(d). The red presents region bigger than the reference model on each model, whereas the blue presents region smaller than reference model on each model. Also the green presents region similar to the reference model on each model, meaning that morphological deviation is close to 0 mm. The results from Figures 9(a), 9(b), and 9(c) can be quantitatively shown as Figure 9(e). The graph of Figure 9(e) shows the number of nodes to compare the deviation between two models. The percentage (%) in graph indicates the deviation distribution per section. It is shown that the model becomes very similar to the reference model as the green area in Figures 9(a), 9(b), and 9(c) and the node numbers close to 0 mm in Figure 9(e) are increased. Considering that the mean distance of (a) is 0.0388 mm, (b) is 0.1081 mm, and (c) is 0.0117 mm, Method 4 was the most similar in shape to Method 1. Additionally, considering that the deviation gap distribution from 0.0117 mm to 0.2505 mm is 52.38% and that from −0.227 mm to 0.117 mm is 30.85%, we can deduce that Method 4 is very similar to Method 1 as 83.23% of the total deviation is less than 0.227 mm.

### 3.2. Comparison of the FE Analysis of the Models Where the Sectionalized Algorithm Was Applied

As the shape of the model applied to each algorithm affects the interpretation result, we analyzed the changes in ACL tension from bending movements of the knee. We measured the von Mises stress, reaction force, and contact stress at 0°, 45°, 90°, and 135° in order to compare the interpretation results from the reference model (Method 1) with those of the models (Methods 2, 3, and 4) from the application of each algorithm. Moreover, the same ACL model and tension were applied in identical environments to match the conditions. We compared the error rate of Method 1 to that of Methods 2, 3, and 4.

**Figure 10:**
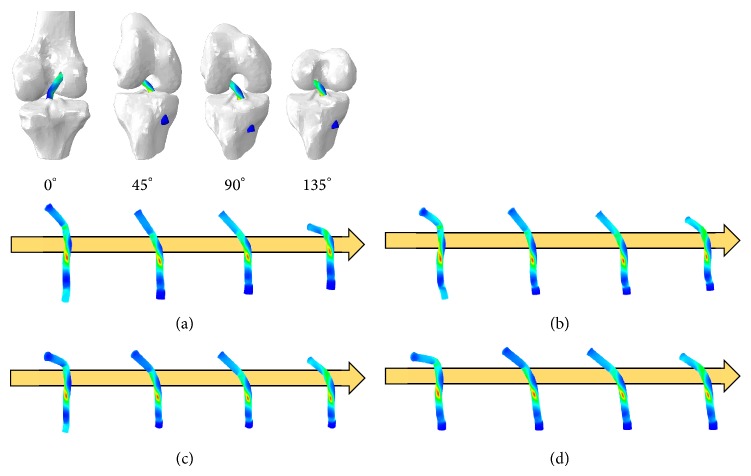
von Mises stress of the simulation results for the models using (a) Method 1, (b) Method 2, (c) Method 3, and (d) Method 4.

As von Mises stress is the sum of all stresses damaging the ACL bundle from flexing the knee, we measured the stress occurring at each angle by selecting the node where stress distribution occurs most significantly in the ACL bundle. The simulation results from each model under equivalent stress are shown in Figure 10.

**Figure 11:**
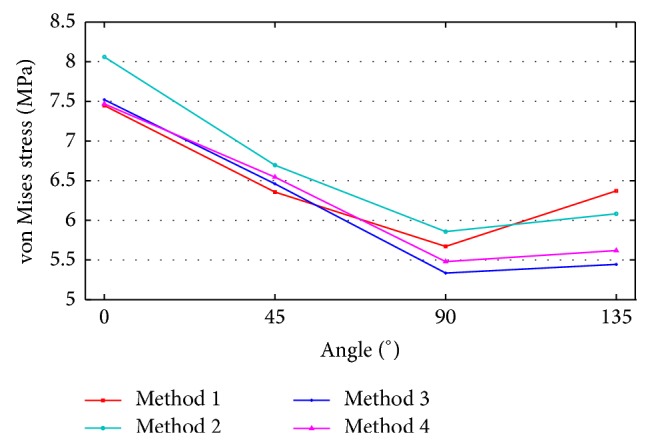
Changes in the von Mises stress of models at different angles.

**Figure 12:**
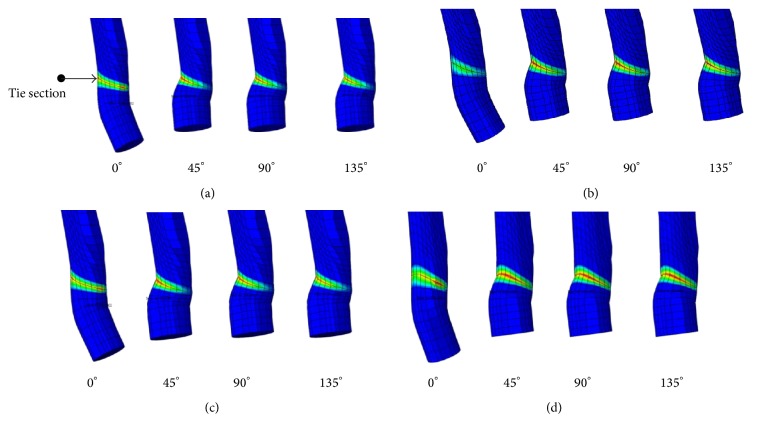
Reaction force simulation result for the tied sections of the models: (a) Method 1, (b) Method 2, (c) Method 3, and (d) Method 4.

**Figure 13:**
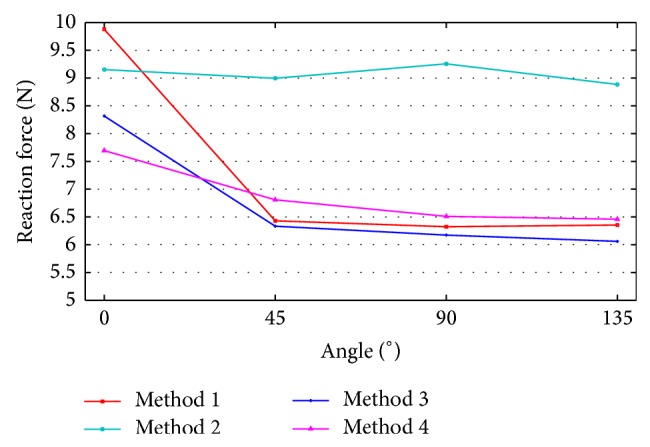
Reaction force changes in the tied sections of the models.

According to the analysis results, stress was represented in the middle of the ACL by movement of the femur. A quantitative comparison of stress distribution results based on the node of strongest stress is shown in Figure 11. The von Mises stress analysis results show that the stress is deceased from 0 to 90° and increased from 90 to 135°. Method 4 was based on the von Mises stress error rate of 1.152 and was similar to Method 1. The ACL bundle between the femur and tibia has a tension of 30 N, and its elasticity added credibility to the analysis results. The reaction force on the tied section of bone was caused by this tension, and it was used to analyze the ACL through femur movement.  Figure 12 shows the reaction force results from the four algorithms. We obtained the data shown in Figure 13 from a quantitative comparison of analysis results from the tied region. According to the reaction force graph, algorithms other than those of Method 2 showed a tendency to decrease reaction force in response to movement of the femur. Method 3, based on a reaction force error rate of 2.1028, was similar to Method 1.

**Figure 14:**
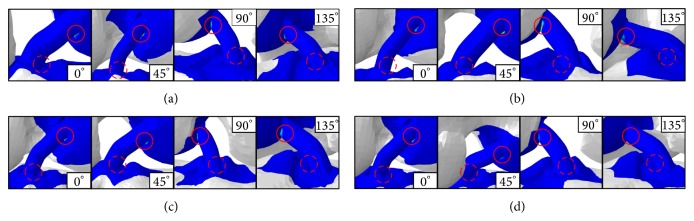
Contact regions and analysis results between bone and ACL: (a) Method 1, (b) Method 2, (c) Method 3, and (d) Method 4.

**Figure 15:**
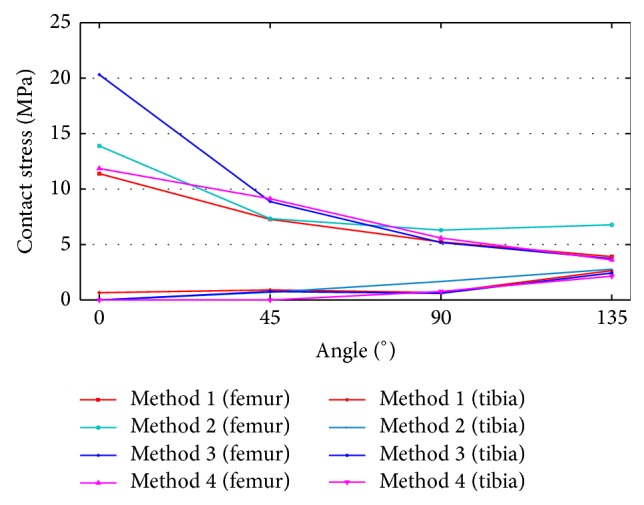
Contact stress changes in the models between bone and ACL.

Measurements between the ACL bundle and the bone tunnel (both femur and tibia) with flexed knee are shown in Figure 14.  Figure 15 shows the quantitative analysis results for contact stress. Contact stress between the femur and ACL decreased, as shown in Figure 15, whereas contact stress between the tibia and ACL increased. This result was determined in the same way as in the models that applied to the other algorithms. Method 4 had an error rate of 2.966 and was similar to Method 1 in contact stress between the femur and ACL. Method 3 had an error rate of 1.192 and was similar to Method 1 in contact stress between the tibia and ACL.

Table 1 summarizes the comparison results for von Mises stress, the reaction force, and the contact stress obtained using the 3 methods.

We applied the root mean squares (RMS) error as shown in (10) to examine the error rate. Consider

(10) $$\begin{array}{c} E_{\text{RMS}}=\sqrt{\frac{1}{4}\overset{n}{\underset{i}{\sum }}{\left({\text{TM}}_{i}-{\text{RM}}_{i}\right)}^{2}},\;\text{for}\;\;i=0,45,90,135, \end{array}$$

where *n* implies the flexion angles of the femur defined as 0, 45, 90, and 135 degrees. RM_*i*_ and TM_*i*_ are defined as the reference method (Method 1) and the compared methods (Methods 2, 3, and 4) with the predefined angles, respectively.

Method 4 had an error rate comparable to that of Method 1 (*α*_1_: 1.428, *α*_2_: 1.439, *α*_3_: 1.152) based on von Mises stress. Additionally, Method 3 had an error rate comparable to that of Method 1 (*α*_1_: 8.754, *α*_2_: 2.1028, *α*_3_: 2.85) based on the reaction force. Method 4 had an error rate comparable to that of Method 1 (*α*_1_: 6.487, *α*_2_: 10.756, *α*_3_: 2.966) based on contact between the femur and ACL. Method 3 had an error rate comparable to that of Method 1 (*α*_1_: 2.227, *α*_2_: 1.192, *α*_3_: 2.409) based on contact between the tibia and ACL. It was shown that the Canny edge detection algorithm (Method 4) was optimal for solid model knee joint reconstruction in the results of 3 out of 5 experiments, as shown in Table 1.

## 4. Conclusions

With high demand for image segmentation to generate 3D model from CT data, interest in objective assessment on the 3D models obtained using various segmentation methods has been increasing. Objective comparison and FE analysis were introduced to find the most suitable segmentation to create a 3D model of the knee joint CT images in this paper. We analyzed the stress distributions and morphological errors in 3D reconstruction by using edge segmentation algorithms. 3D solid models were reconstructed by three types of algorithms typically used in the field of image processing.

What this study found can be summarized as the following.

- From the morphological deviations and FE analysis results (von Mises stress, reaction force, and contact stress) of the ACL graft compared, it was proven that it is possible to obtain reliable data from image processing algorithms without the aid of a clinician.
- The morphological comparison results from the experiment showed that 83.23% of all nodes showed a deviation of less than 0.227 mm.
- In measurements of the von Mises stress, reaction force, and contact stress by FE analysis, Method 4 and Method 3 showed lower error rates for von Mises stress and reaction force, respectively.
- For contact stress, Method 4 and Method 3 showed lower contact error rates for femur-ACL stress and tibia-ACL stress, respectively.

In conclusion, the Canny edge detection algorithm (Method 4) showed good performance in the results of 3 out of 5 experiments, demonstrating that it is optimal for the reconstruction of a 3D solid model (Method 4, Method 3, and Method 2 in order).

## Figures and Tables

**Table 1:** Error rates between Method 1 and Methods 2, 3, and 4.

Interpretation type	0°	45°	90°	135°	*η*
von Mises stress (MPa)					
Method 1	7.447	6.356	5.67	6.371	—
Method 2	8.06	6.694	5.858	6.082	1.428
Method 3	7.520	6.460	5.335	5.444	1.439
Method 4	7.468	6.544	5.479	5.619	1.152
Reaction force (N)					
Method 1	9.878	6.43	6.324	6.354	—
Method 2	9.153	8.996	9.256	8.885	8.754
Method 3	8.317	6.333	6.1732	6.06	2.103
Method 4	7.695	6.808	6.510	6.459	2.852
Contact stress (MPa)					
Method 1					
Femur	11.391	7.278	5.235	3.914	—
Tibia	0.66	0.907	0.432	2.651	—
Method 2					
Femur	13.89	7.335	6.302	6.778	6.487
Tibia	0	0.7	1.669	2.774	2.227
Method 3					
Femur	20.319	8.87	5.185	3.728	10.756
Tibia	0	0.765	0.606	2.435	1.192
Method 4					
Femur	11.853	9.128	5.587	3.612	2.966
Tibia	0	0	0.77	2.147	2.409

(*α*_1_: Method 2 − Method 1, *α*_2_: Method 3 − Method 1, *α*_3_: Method 4 − Method 1, η: error rate by (10)).
